# Are people living with HIV have a low vulnerability to omicron variant infection: results from a cross-sectional study in China

**DOI:** 10.1186/s12879-023-08768-x

**Published:** 2023-11-14

**Authors:** Yuting Tan, Songjie Wu, Wei Guo, Jie Liu, Fangzhao Ming, Shi Zou, Weiming Tang, Ke Liang, Junjun Yang

**Affiliations:** 1 Department of Infectious Diseases, Zhongnan Hospital of Wuhan University, Wuhan, 430071 China; 2 Wuhan Research Center for Infectious Diseases and Cancer, Chinese Academy of Medical Sciences, Wuhan, China; 3 Department of Nosocomial Infection Management, Zhongnan Hospital of Wuhan University, Wuhan, China; 4 Department of Pathology, Zhongnan Hospital of Wuhan University, Wuhan, China; 5 Department of Pathology, School of Basic Medical Sciences, Wuhan University, Wuhan, China; 6 Wuchang District Center for Disease Control and Prevention, Wuhan, China; 7 University of North Carolina Project-China, Guangzhou, China; 8 Hubei Engineering Center for Infectious Disease Prevention, Control and Treatment, Wuhan, China; 9 Jiangnan University Medical Center, Wuxi, 214122 China

**Keywords:** HIV, Omicron, Epidemiology, Risk factors, COVID-19 vaccines

## Abstract

**Background:**

A surge of more than 80 million Omicron variant infected cases was reported in China less than a month after the "zero COVID" strategy ended on December 7, 2022. In this circumstance, whether people living with HIV (PLWH) in China experience a similar risk is not clear.

**Methods:**

A cross-sectional study was conducted in the Wuchang District of Wuhan between December 20, 2022, and January 18, 2023 through a self-administered online survey. PLWH and HIV-negative people aged ≥ 18 years old who volunteered for this survey were eligible. The prevalence of Omicron variant infection between PLWH and HIV-negative people was compared, and the factors associated with the Omicron variant infection among PLWH and HIV-negative people were further evaluated, respectively.

**Results:**

In total, 890 PLWH and 1,364 HIV-negative adults from Wuchang District were enrolled. Among these participants, 690 PLWH (77.5%) and 1163 HIV-negative people (85.3%) reported SARS-CoV-2 infection. Gender, chronic disease conditions, and COVID-19 vaccination status significantly differed between the two groups. After adjusting gender, age, comorbidities, and COVID-19 vaccination status, the risk of SARS-CoV-2 infection among PLWH was significantly lower than among HIV-negative people (aOR 0.56, 95%CI 0.42–0.76). Multivariable logistic regression analysis showed that PLWH with older age and detectable HIV-viral load (HIV-VL) had decreased risk of SARS-CoV-2 infection (aOR 0.98, 95%CI 0.96–0.99; aOR 0.59, 95%CI 0.36–0.97). Compared with PLWH receiving one/two doses of COVID-19 vaccines, no significant differences in the risk of SARS-CoV-2 infection were observed among PLWH receiving three doses of inactivated vaccines and four doses of vaccines (three doses of inactivated vaccines plus one dose of inhaled recombinant adenovirus type 5 (AD5)-vectored vaccine). Among HIV-negative people, those receiving four doses of COVID-19 vaccines had a lower risk of SARS-CoV-2 infection than those receiving one/two doses (aOR 0.14, 95%CI 0.08–0.25).

**Conclusions:**

Our study proves that PLWH have a lower risk of Omicron variant infection than HIV-negative people. However, even PLWH with younger age and virological suppression should strengthen the prevention against SARS-CoV-2 infection. Three doses of inactivated vaccines plus one dose of inhaled recombinant AD5-vectored COVID-19 vaccine may provide better protection for HIV-negative people.

## Background

The "zero COVID" strategy” has been implemented for more than two years in China, which effectively controls the spread of the virus in China. Under the goal of maintaining the necessary level of economic activity and restraining virus outbreaks, the 20 measures on November 1, 2022 [1] and further the 10 measures on December 7, 2022 [2] were announced. Since the ending of "zero COVID" strategy on December 7, 2022 during the Omicron wave, restricting the detection coverage, shortening the isolation period of close contacts or inbound travelers, suspending the tracking of secondary contacts, prohibiting large-scale detection within the region, and allowing family isolation or quarantine have created opportunities for the rapid spread of the virus. A nationwide pandemic of the Omicron variant has been ongoing in China including Hubei Province [3, 4]. According to the World Health Organization (WHO) statistics, China has experienced a surge of more than 80 million cases of the Omicron variant infection in less than a month, and nearly 40,000 new confirmed deaths during this period [5]. Take Beijing, one of the most crowded cities in China, as an example, the cumulative infection attack rate is estimated to reach 76% on December 22, 2022 [6].

The data on the susceptibility to SARS-CoV-2 infection among people living with HIV (PLWH) remains controversial [7–9]. Previous studies reported that PLWH had increased COVID-19 mortality compared to the general people due to their immunosuppressed state and high rate of comorbidities [10–12]. Even though a series of studies have described the clinical outcomes of COVID-19 among PLWH, little is known about whether PLWH are more vulnerable to the Omicron variant infection in China. And the removal of the "zero COVID" strategy in China provided a unique opportunity to study this.

In this circumstance, we conducted a cross-sectional study to assess the risk of the Omicron variant infection among PLWH by comparison with HIV-negative people. We further explored the potential risk factors for the Omicron variant infection among PLWH and HIV-negative people.

## Methods

### Study participants

Between December 20, 2022, and January 18, 2023, a cross-sectional study with an online self-administered questionnaire was conducted in Wuchang District, Wuhan, Hubei Province. All PLWH managed by the Wuchang district center for disease control and prevention (CDC), and HIV-negative people from the general population in Wuchang District were reached out for the survey by distributing the questionnaire via the Wenjuanxing of WeChat, a questionnaire platform. The inclusion criteria of PLWH and HIV-negative people included: 1) volunteered to participate in the survey; 2) age ≥ 18 years old.

### Questionnaire and data collection

Two separate online self-administered questionnaires were designed for PLWH and HIV-negative people independently. The following data were collected from PLWH and HIV-negative people: gender, age, diabetes (diabetes, none), cardiovascular and cerebrovascular diseases (cardiovascular and cerebrovascular diseases, none), chronic lung diseases (chronic lung diseases, none), chronic kidney diseases (chronic kidney diseases, none), chronic liver diseases (chronic liver diseases, none), COVID-19 vaccination status (unvaccinated, or one/two doses, or three doses, or four doses (three doses of inactivated vaccines plus one dose of inhaled recombinant adenovirus type 5 (AD5)-vectored COVID-19 vaccine, Convidecia), current SARS-CoV-2 infection status (positive SARS-CoV-2 nucleic acid testing, or positive SARS-CoV-2 rapid antigen test, or uninfected). For PLWH, data on antiretroviral therapy (ART) status (on ART, or none), current ART regimen (non-nucleoside reverse transcriptase inhibitor (NNRTI), or lopinavir/ritonavir (LPV/r), or integrase strand transfer inhibitors (INSTIs)), recent CD4^+^ T lymphocyte count (CD4 count, (0–199 cells/µL, or 200–349 cells/µL, or 350–499 cells/µL, or ≥ 500 cells/µL, or unknown)), and HIV viral load (HIV-VL, undetectable (< 20 IU/ml), detectable (≥ 20 IU/ml), unknown) were also collected.

The diagnosis of SARS-CoV-2 infection depended on the self-reporting of positive SARS-CoV-2 nucleic acid testing or positive rapid antigen test by participants [13].

### Statistical analysis

SPSS version 26·0 (IBM Corp, Armonk, New York, United States) was used for data analysis. Normally distributed continuous variables were presented as mean ± standard deviation (SD), while continuous variables without normal distribution were presented as median and the 25th to 75th interquartile range (IQR). Categorical variables were denoted as counts and proportions (%). Group t-test or non-parametric rank sum test was used for the analyses of continuous variables, and the chi-square test or Kruskal–Wallis rank sum test was used for the analyses of counts data. Multivariable logistic regression analysis was performed to evaluate the factors associated with SARS-CoV-2 infection among all the study participants (PLWH and HIV-negative people), by adjusting the confounding variables, including gender, age, different comorbidities, and COVID-19 vaccination status. For the analysis of risk factors associated with SARS-CoV-2 infection among PLWH, all variables, including gender, age, various comorbidities, COVID-19 vaccination status, ART status, ART regimen, CD4 count, and HIV viral load, were included in the multivariable logistic regression models. Variables, including gender, age, different comorbidities, and COVID-19 vaccination status, were included in the multivariable logistic regression analysis of risk factors associated with SARS-CoV-2 infection among HIV-negative people. A two-sided p-value < 0.05 was considered statistically significant.

## Results

### Participants’ characteristics

Among 1,064 PLWH in Wuchang District (all aged ≥ 18 years old), 962 volunteered to participate in the survey, and 890 (92.5%) PLWH finished the survey. In addition, 1,478 HIV-negative adults from the Wuchang District volunteered to participate in the survey. Of them, 1,412 were aged ≥ 18 years old, and 1,364 (92.3%) finished the survey.

As shown in Table 1, the proportions of males (84.6% vs. 27.5%, p < 0.001) and people with chronic liver diseases (4.7% vs. 2.1%, *p* = 0.001) were significantly higher among PLWH than among HIV-negative people. The distributions of age and other chronic diseases had no significant difference between the two groups. Compared with HIV-negative people, the proportion of three doses of COVID-19 vaccination was higher (73.6% vs. 64.8%), and the proportion of four doses of COVID-19 vaccination was lower among PLWH (1.0% vs. 8.2%).

**Table 1:** Characteristics of PLWH and HIV-negative people enrolled in the study (*N* = 2,254)

	PLWH (*n* = 890)	HIV-negative (*n* = 1364)	*p* value
Male	753 (84.6)	375 (27.5)	< 0.001
Age, years, median (IQR)	36 (30–47.25)	36 (31–45)	0.613
COVID-19 vaccination, n (%)			< 0.001
One dose/two doses	166 (18.6)	282 (20.7)	
Three doses	655 (73.6)	884 (64.8)	
Four doses^a^	9 (1.0)	112 (8.2)	
Unvaccinated	60 (6.7)	86 (6.3)	
Diabetes, n (%)	27 (3.0)	34 (2.5)	0.507
Cardiovascular and cerebrovascular diseases, n (%)	73 (8.2)	129 (9.4)	0.327
Chronic lung diseases, n (%)	26 (2.9)	31 (2.3)	0.340
Chronic kidney diseases, n (%)	12 (1.3)	17 (1.2)	0.850
Chronic liver Diseases, n (%)	42 (4.7)	29 (2.1)	0.001
CD4 T count [cells/µL, n (%)]			
0–199	71 (8.0)	··	··
200–349	144 (16.2)	··	··
350–499	203 (22.8)	··	··
≥ 500	332 (37.3)	··	··
Unknown	140 (15.7)	··	··
HIV viral load, n (%)			
Undetectable	620 (69.6)	··	··
Detectable	105 (11.8)	··	··
Unknown	165 (18.5)	··	··
On ART, n (%)	856 (96.2)	··	··
ART regimens, n (%)			
None	34 (3.8)	··	··
NNRTI	464 (52.1)	··	··
LPV/r	65 (7.3)	··	··
INSTIs	377 (42.3)	··	··

*PLWH* People living with HIV, *CD4 count* CD4^+^ T lymphocyte count, *ART* Antiretroviral therapy, *NNRTI* Non-nucleoside reverse transcriptase inhibitor, *LPV/r* Lopinavir/ritonavir, *INSTIs* Integrase strand transfer inhibitors

^a^refers to three doses of inactivated COVID-19 vaccines plus one dose of recombinant adenovirus type 5 (AD5)-vectored COVID-19 vaccine, Convidecia. Data are median (IQR) or n (%)

Overall, 690 PLWH (77.5%) and 1163 HIV-negative people (85.3%) in the Wuchang District reported SARS-CoV-2 infection between December 20, 2022, and January 18, 2023, and the rate of SARS-CoV-2 infection in the former was significantly lower (*p* < 0.001).

### Factors associated with SARS-CoV-2 infection among all the study participants

As shown in Table 2, among all 2,254 individuals, older age (aOR 0.98, 95%CI 0.97–0.99) and receiving four doses of COVID-19 vaccines (aOR 0.18, 95%CI 0.11–0.28) were associated with lower risk of the Omicron variant infection. In addition, females have an increased risk of the Omicron variant infection (aOR 1.39, 95%CI 1.05–1.83).

**Table 2:** Risk factors associated with SARS-CoV-2 infection among PLWH and HIV-negative people (*N* = 2,254)

	aOR (95%CI)	*p* value
Sex		
Male (ref)		
Female	**1.39 (1.05–1.83)**	**0.019**
Age, years	**0.98 (0.97–0.99)**	**0.005**
HIV infection	**0.56 (0.42–0.75)**	** < 0.001**
COVID-19 vaccination		
One dose/two doses (ref)		
Three doses	1.02 (0.75–1.38)	0.879
Four doses^a^	**0.18 (0.11–0.28)**	** < 0.001**
Unvaccinated	0.65 (0.40–1.06)	0.089
Diabetes	0.86 (0.45–1.65)	0.662
Cardiovascular and cerebrovascular diseases	0.92 (0.58–1.46)	0.745
Chronic lung diseases	1.09 (0.52–2.28)	0.811
Chronic kidney diseases	0.51 (0.21–1.23)	0.137
Chronic liver Diseases	0.78 (0.41–1.46)	0.443

^a^refers to three doses of inactivated COVID-19 vaccines plus one dose of inhaled recombinant adenovirus type 5 (AD5)-vectored COVID-19 vaccine, Convidecia

After adjusting the confounding factors, including gender, age, comorbidities, and COVID-19 vaccination status, the risk of the Omicron variant infection among PLWH was significantly lower than among HIV-negative people (aOR 0.56, 95%CI 0.42–0.76).

### Factors associated with SARS-CoV-2 infection among PLWH

**Fig. 1:**
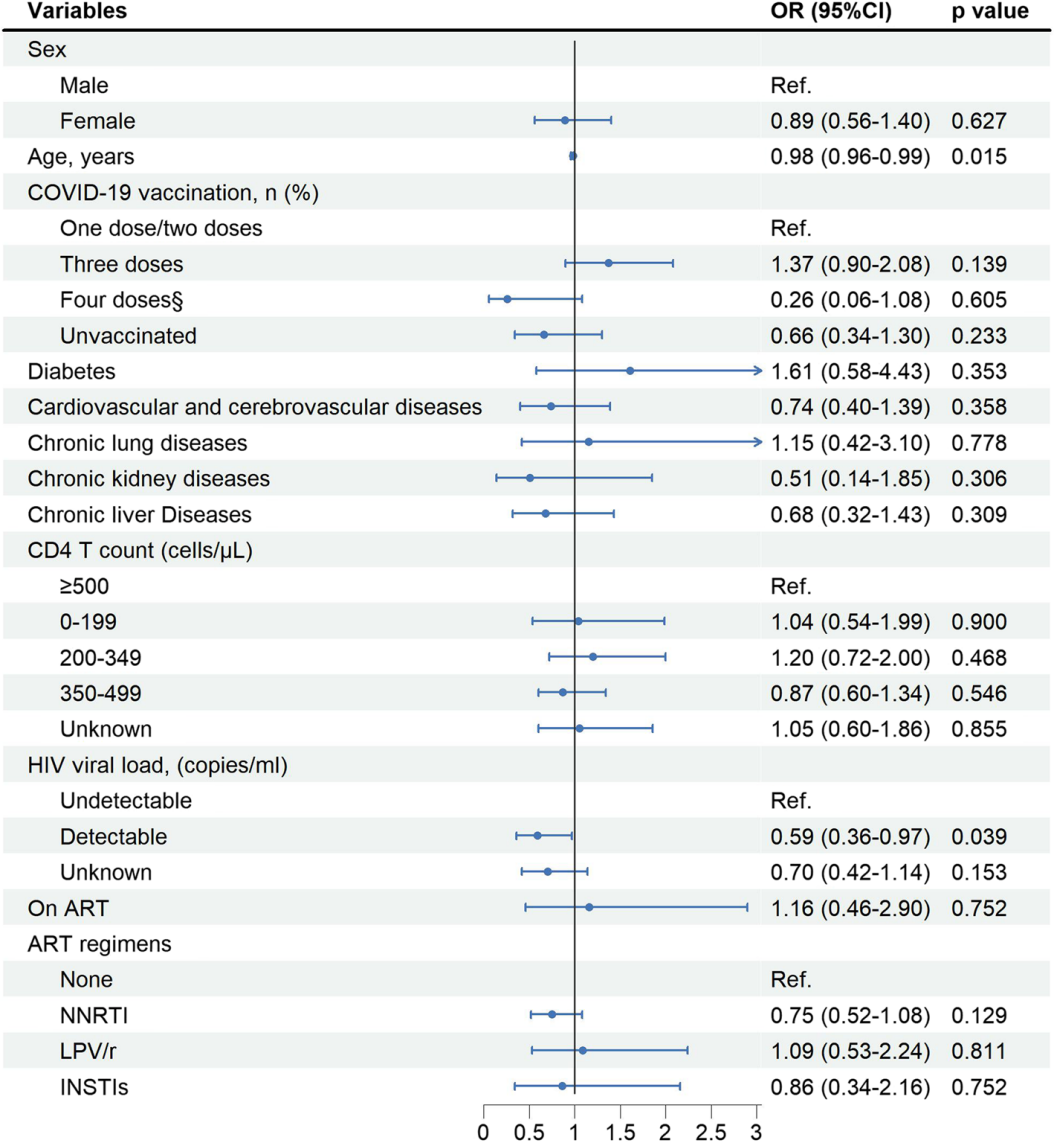
Risk factors associated with SARS-CoV-2 infection among PLWH (*N* = 890). ^§^refers to three doses of inactivated COVID-19 vaccines plus one dose of inhaled recombinant adenovirus type 5 (AD5)-vectored COVID-19 vaccine, Convidecia. PLWH = people living with HIV. CD4 count = CD4^+^ T lymphocyte count, ART = antiretroviral therapy. NNRTI = non-nucleoside reverse transcriptase inhibitor, LPV/r = lopinavir/ritonavir, INSTIs = integrase strand transfer inhibitors

PLWH with detectable HIV-VL had a lower risk of SARS-CoV-2 infection than those with undetectable HIV-VL (aOR 0.59, 95%CI 0.36–0.97). Compared with PLWH receiving one/two doses of COVID-19 vaccines, there is no significant difference in the risk of SARS-CoV-2 infection among PLWH receiving three doses of COVID-19 vaccines and among PLWH receiving four doses of COVID-19 vaccines. In addition, having different comorbidities on ART, different ART regimens, and CD4 count had no significant association with the risk of SARS-CoV-2 infection (Fig. 1).

### Factors associated with SARS-CoV-2 infection among HIV-negative people

**Fig. 2:**
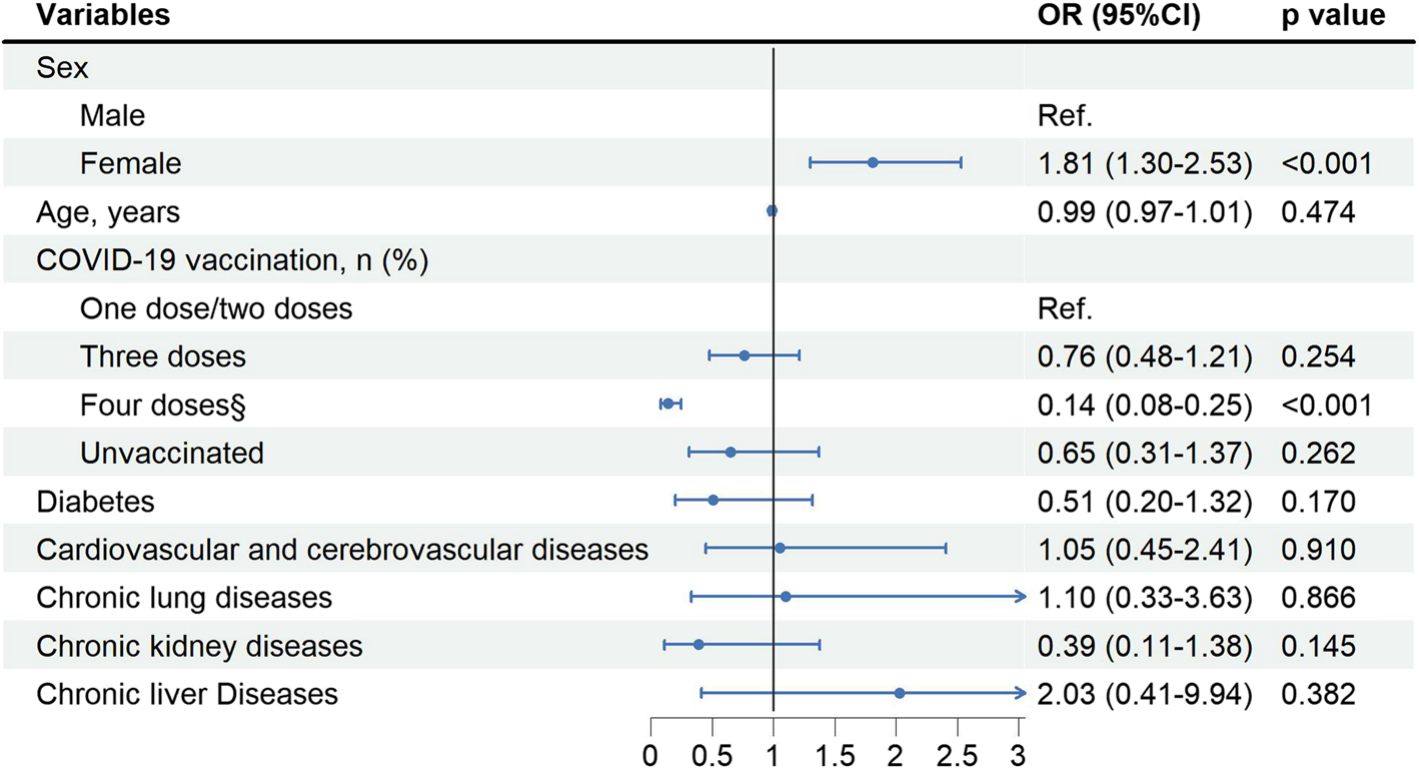
Risk factors associated with SARS-CoV-2 infection among HIV-negative people (*N* = 1,364). ^§^refers to three doses of inactivated COVID-19 vaccines plus one dose of inhaled recombinant adenovirus type 5 (AD5)-vectored COVID-19 vaccine, Convidecia

As shown in Fig. 2, female participants have an increased risk of SARS-CoV-2 infection than male participants (aOR 1.81, 95%CI 1.30–2.53). Compared to HIV-negative people receiving one/two doses of COVID-19 vaccines, those receiving four doses of COVID-19 vaccines had a decreased risk of SARS-CoV-2 infection (aOR 0.14, 95%CI 0.085–0.25).

## Discussion

Realizing the risk and risk factors associated with the Omicron variant infection among PLWH is essential for COVID-19 management in China. By filling this knowledge gap, our study found that PLWH had a significantly lower prevalence of Omicron variant infection than HIV-negative people, and PLWH with older age and detectable HIV-VL had decreased risk of SARS-CoV-2 infection.

We found that the prevalence of Omicron variant infection was lower among PLWH than HIV-negative adults. A prospective observational cohort study including 5683 PLWH on ART in Barcelona showed that the standardized incidence rate ratios of confirmed or confirmed/probable COVID-19 in PLWH were 38% (95%CI 27%-52%) and 33% (95%CI 21%-50%), respectively relative to the general population [14]. Another prospective cohort study conducted in Spain evolving 77,590 HIV-positive persons receiving ART estimated that the age- and sex-standardized risk of confirmed COVID-19 infection among PLWH (30/10,000) was lower compared to the general population (41.7/10,000) [15]. All these data was consistent with our data that PLWH had a lower incidence of SARS-CoV-2 infection than the general population. One possible explanation is that asymptomatic SARS-CoV-2 infection may be more common among PLWH than those without HIV [16], and some PLWH with asymptomatic infection may not be captured and remain undiagnosed. The another explanation is that ART may be associated with the lower incidence of SARS-CoV-2 infection. Studies have found that HIV-infected patients receiving tenofovir disoproxil fumarate (TDF)-based regimens had lower risk for COVID-19 diagnosis [17]. Additionally, PLWH may be more cautious and vigilant about COVID-19 prevention measures than those without HIV, especially after adjusting the "zero COVID" strategy in China [9, 10].

This study did not observe an increased risk of omicron variant infection among PLWH with lower CD4 counts. At the same time, PLWH with detectable HIV-VL had a decreased risk of infection. Previous studies reported that neither the CD4 count nor HIV viral load was associated with the incidence of COVID-19 in PLWH, suggesting that PLWH with immunosuppression may have no increased risk of SARS-CoV-2 infection [18–20]. However, most PLWH enrolled in these studies reached virological suppression, so the association between HIV-VL and the risk of SARS-CoV-2 infection should be considered cautiously. Whether CD4 count and HIV-VL play a role in SARS-CoV-2 infection needs further prospective investigation among PLWH.

Our study suggested that three doses of inactivated COVID-19 vaccination did not prevent omicron variant infection than one/two doses of inactivated vaccination among either PLWH or HIV-negative people. Although growing evidence showed that the third dose of COVID-19 vaccine could induce rapid and stronger humoral immune responses against SARS-CoV-2 among PLWH and those without HIV [21–23], a protective effect was not found for omicron variant infection among both PLWH and HIV-negative people enrolled in our study. One potential reason is that the protective effect of the third dose of inactivated COVID-19 vaccine nay have waned since participants completed their three-dose regimen one year ago [24]. The second potential reason is that most participants received inactivated COVID-19 vaccines that were not specifically targeted against the Omicron variant. For example, a study performed in Shanghai during the Omicron BA.2 wave even found that the nAb titers against the Omicron variants were significantly lower in individuals receiving three doses of vaccines than in individuals receiving two doses of vaccines [25]. Hence, in the current situation, PLWH and HIV-negative people may not benefit from three doses of inactivated COVID-19 vaccination to avoid the omicron variant infection, while vaccines designed to prevent the omicron variant infection are needed.

Compared with one/two doses of COVID-19 vaccination, we indicated that three doses of inactivated vaccination plus one dose of inhaled recombinant AD5-vectored vaccination protects against the Omicron variant infection among HIV-negative people. The effect of four doses of mRNA COVID-19 vaccines on reducing the rate of the Omicron variant infection have been verified by two large real-world studies in Israel [26, 27]. For individuals who had previously received one or two doses of CoronaVac, heterologous boosting with recombinant AD5-vectored vaccine could elicit significantly increased nAbs against SARS-CoV-2 than homologous boosting with CoronaVac [28]. All of those data supported that the fourth dose of inhaled recombinant AD5-vectored vaccination should be implemented to prevent the omicron variant infection.

This study has several limitations that need to take into consideration. First, selection bias may exist in this study, especially for sampling HIV-negative adults. However, we have minimized the bias by randomly distributing the online questionnaire to HIV-negative people and increasing the response rate by improving the quality of the online questionnaire. Second, not all individuals with asymptomatic infection performed the SARS-CoV-2 nucleic acid testing or rapid antigen test, and cases with asymptomatic infection may be missed. Thus, the prevalence of SARS-Co-V-2 infection may be underestimated, especially among HIV-negative people. Third, residual or unmeasured confounding may exist in the risk factors analyses of COVID-19. Moreover, the online questionnaire was distributed to all PLWH managed by Wuchang District Center for Disease Control and Prevention, but not to all HIV-negative people in Wuchang District. Therefore, selection bias existed, which might influence the results to some extent.

## Conclusions

Our study provided the evidence that PLWH have lower prevalence of Omicron variant infection than HIV-negative people, and PLWH with younger age and virological suppression should strengthen the prevention against SARS-CoV-2 infection. To our knowledge, this is the first study to investigate the prevalence of SARS-CoV-2 infection among PLWH by comparison with HIV-negative people during the Omicron wave after the "zero COVID" strategy ended. Our findings warrant further investigation in the interaction between SARS-CoV-2 and HIV, and in the role of ART and immunosuppression to quantify the risks of SARS-CoV-2 infection among PLWH in different situations.

## Data Availability

The original contributions presented in the study are included in the article. Further inquiries can be directed to the corresponding authors.

## Abbreviations

PLWH: People living with HIV
HIV-VL: HIV-viral load
AD5: Adenovirus type 5
WHO: World Health Organization
WHO: World Health Organization
CDC: Center for disease control and prevention
ART: Antiretroviral therapy
NNRTI: Non-nucleoside reverse transcriptase inhibitor
LPV/r: Lopinavir/ritonavir
INSTIs: Integrase strand transfer inhibitors
CD4 count: CD4^+^ T lymphocyte count
IQR: Interquartile range
TDF: Tenofovir disoproxil fumarate
